# Improving Structural MRI Preprocessing with Hybrid Transformer GANs

**DOI:** 10.3390/life13091893

**Published:** 2023-09-11

**Authors:** Ovidijus Grigas, Rytis Maskeliūnas, Robertas Damaševičius

**Affiliations:** 1 Faculty of Informatics, Kaunas University of Technology, 50254 Kaunas, Lithuania; 2 Department of Applied Informatics, Vytautas Magnus University, 44248 Kaunas, Lithuania

**Keywords:** magnetic resonance imaging, super resolution

## Abstract

Magnetic resonance imaging (MRI) is a technique that is widely used in practice to evaluate any pathologies in the human body. One of the areas of interest is the human brain. Naturally, MR images are low-resolution and contain noise due to signal interference, the patient’s body’s radio-frequency emissions and smaller Tesla coil counts in the machinery. There is a need to solve this problem, as MR tomographs that have the capability of capturing high-resolution images are extremely expensive and the length of the procedure to capture such images increases by the order of magnitude. Vision transformers have lately shown state-of-the-art results in super-resolution tasks; therefore, we decided to evaluate whether we can employ them for structural MRI super-resolution tasks. A literature review showed that similar methods do not focus on perceptual image quality because upscaled images are often blurry and are subjectively of poor quality. Knowing this, we propose a methodology called HR-MRI-GAN, which is a hybrid transformer generative adversarial network capable of increasing resolution and removing noise from 2D T1w MRI slice images. Experiments show that our method quantitatively outperforms other SOTA methods in terms of perceptual image quality and is capable of subjectively generalizing to unseen data. During the experiments, we additionally identified that the visual saliency-induced index metric is not applicable to MRI perceptual quality assessment and that general-purpose denoising networks are effective when removing noise from MR images.

## 1. Introduction

Structural magnetic resonance imaging (sMRI) is a widely used medical imaging technique that provides detailed information about the structure of the brain [1]. However, sMRI images are often affected by various types of noise and artifacts, which can reduce the accuracy of subsequent analysis and diagnosis [2]. Preprocessing of sMRI images is an essential step to improve the quality of the images and enhance the accuracy of the analysis. Generally, MRI images have around 1 mm × 1 mm in-plane resolution with 2–3 mm thickness slices. This type of resolution is approximately equal to a 256 × 256 pixel density image when dealing with a slice of a brain on one of the axial, coronal or sagittal planes. With such low resolution, it is hard to distinguish small details, which could be essential for detection of changes in the brain due to some mild pathological disease. To overcome this issue, a super-resolution technology is usually used, which allows upscaling low-resolution images into higher resolution. This technique is particularly useful in clinical practice (for example, in Alzheimer’s disease diagnostics [3]) because magnetic resonance tomographs, which can produce high-quality and high-resolution images, are expensive, and the imaging procedure itself takes a long time. The study [4] demonstrated that AI models could learn complex clinical information from photos and differentiate tumor slides. Modern computer vision processing techniques [5] with combined models were able to correctly extract data from the first encoder–decoder network and merge it with the second encoder–decoder network to offer exact anatomical structure segmentation, supporting clinicians in identifying different pulmonary and heart disorders.

Single-image super resolution (SISR) became popular with the release of the super-resolution convolutional neural network (SRCNN) [6] in early 2015, this being one of the first applications of deep convolution neural networks to this problem. Since then, a lot of research has emerged in the field aiming to improve the visual quality of upscaled images. Before the introduction of transformers [7], the majority of solutions used some type of convolutional neural network (CNN). After the adoption of self-attention—the main idea for transformers for images [8,9]—vision transformer (ViT) was introduced, and since then, it has been possible to achieve state-of-the-art results in the single-image super-resolution task.

Looking at more recent work in this field, we can find ViT models, such as HAT [10] and SwinIR [11], and a couple of CNN models, such as Real-ESRGAN [12], BSRGAN [13] and CARN [14], which compete for SOTA performance. The majority of them use the adversarial training techniques first proposed by [15], which make it possible to improve the upscaled images’ visual quality even more.

MR images are 3D volumes that contain brain slices along the sagittal, coronal and axial planes captured by the MR tomograph during the scanning procedure. During signal capture, magnetic field frequencies are measured and encoded into so called “k” space, which is the frequency domain. After applying a Fourier transform to the frequency domain, we get an image where the signal intensity is now represented by pixel brightness. This approach is applied for each spatial component (“voxel”) in 3D space. Since MR images are 3D objects, the majority of applications use 3D network architectures to manage this. For example, the authors of [16] used ResNet to upscale T2w MR images, and they also used T1w as a reference to further improve the quality of the upscaled T2w image with another ResNet network. In another example [17], the authors used a combination of a CNN and ResNet to upscale 3D MR images. The main difference was that they utilized adjacent MRI slices in the network layers. There have also been attempts to divide 3D volume into patches and then learn filters that are capable of upscaling patches, which can be combined back with the whole volume at the end, as described in [18]. We can also find other methods, such as deep 3D CNNs with skip-connections, like in [19] or [20].

Training and deploying models working with 3D volumes requires more computational power and VRAM. It is more efficient to work in 2D space with slices from a 3D volume, since when doctors need to evaluate MRI in search of any pathologies, they usually look at a collection of slices for each plane. There are a few examples, such as [20] or [21], where researchers have tried to upscale 2D MR images with a U-Net model or with classical ResNet [22]. There is also a study [23] where, after upscaling 2D slices, the slices were combined back into a 3D volume. The authors claimed it is more efficient to work with slices than with volumes.

Another problem with MR images is that they are noisy by nature due to signal interference, low amounts of coils or equipment wear-out. While noise is not a limitation for clinical diagnosis, it can be a roadblock for AI solutions, since networks need to learn the most important features for classification or other tasks, but noise could overwhelm those features. A typical solution for noise reduction is to use a filter, like non-local means [24], anisotropic diffusion [25] or a bilateral filter [26]; however, after application of these filters, MR images become blurry and their perceptual quality decreases.

A common problem in super-resolution and denoising methods is that these methods do not focus on preserving the perceptual quality of images and the evaluation metrics that are used, like the peak-signal-to-noise ratio (PSNR), do not evaluate this aspect of quality. Therefore, the need for methods that preserve the perceptual quality of MR images still remains. We were able to find only one example focused on the perceptual quality of MR images in the recent paper [21], which reported on a perceptual quality metric called learned perceptual image patch similarity (LPIPS) together with PSNR and others. This means the research gap in the field still exists.

In this work, we employed SOTA super-resolution and denoising networks to perform MR image improvements focused on multiple quality aspects, such as pixel-level, style-level and perceptual-level aspects. The novelty and the main contribution of this work are represented by the improvement of the existing state-of-the-art single-image super-resolution method to preserve perceptual quality in MR images, with another state-of-the-art denoising network additionally employed to further improve the quality of upscaled MR images. We call our proposed hybrid method HR-MRI-GAN. The primary reason for concentrating on structural MRI (sMRI) preprocessing was to improve the quality and usability of the images for future analysis and diagnosis in medical contexts [27]. Structural MRI is a key imaging technology used to non-invasively examine the anatomy of the brain and other organs. However, raw MRI images frequently contain noise, abnormalities and defects that might impair interpretation and the accuracy of any subsequent analysis or diagnosis. Preprocessing approaches are thus used to improve the quality of these pictures and make them more acceptable for medical use.

## 2. Related Work

To identify similar research work, we queried two databases: Web of Science and Scopus. We constructed search queries with Boolean operations (AND, OR, NOT) and used these keywords: brain, MRI, upscal*, denois*, super*, preproc*, segm*. We used an asterisk (*) to include all different styles of the same words; for example, denoise or denoising, etc. All the included sources were from scientific journals or conference proceedings and published after 2014. Initially, 116 sources were identified; after deduplication, 91 sources were left. Then, we filtered all identified sources by evaluating their relevance to our solution based on titles and abstracts. After initial filtering, we were left with 26 publications. After a further eligibility study, only four sources were identified that were different from our solution but worth mentioning, as they related to the problem we were solving.

Wu et al. [21] modified the U-Net model architecture and added self-attention layers. They called the model architecture the denoising diffusion probabilistic model (DDPM). The authors focused on very-low-resolution images, as their input into the model only used a 16 × 16 resolution and they performed ×8 upscaling on the Amsterdam open MRI [28] dataset.

Feng et al. [20] combined the U-Net architecture model with a traditional CNN where, in the first part, many upsample/downsample layers are stacked and, deeper in the network, many convolution, pooling and batch normalization layers are stacked. They also used residual connections to share weights with the deeper layer, as this allows sharing of the knowledge between layers. The authors called this method the coupled-projection approach. They used ×4 upscaling on The Cancer Genome Atlas (TCGA) [29] and Anatomical Tracings of Lesions After Stroke (ATLAS) [30] datasets.

Hongtao et al. [23] used an earlier version of Real-ESRGAN [12]—ESRGAN [31]—to upscale slices of brain images and then interpolated all slices back into 3D object. They used ×2 upscaling but did not report the dataset with which they tested their solution. More details on all identified similar works are mentioned in Table 1.

Song et al. [22] used a very similar approach to ours in terms of super resolution; however, they used a more classic network architecture, the residual convolutional neural network, and applied it for the human fetal brain super-resolution task. They used ×2 upscaling with the Kirby 21 [32] and NAMIC [33] datasets.

In summary, these papers include studies on multi-parametric neuroimaging reproducibility, deep learning methods for pixel-level crack detection, perceptual losses for real-time style transfer and super resolution [34,35,36,37], and practical unified motion and missing data treatment with degraded video.

**Table 1:** Existing papers in the field of deep learning that used super-resolution technology to enhance the quality of structural MRI.

Ref.	Input Resolution	Output Resolution	PSNR (dB)	SSIM	Dataset	Model
[21]	16 × 16 (×8)	128 × 128	24.63	0.784	Amsterdam open MRI [28]	U-Net with self-attention
[20]	60 × 60 (×4)	240 × 240	TCGA (36.98), ATLAS (29.02)	TCGA (0.996), ATLAS (0.951)	TCGA [29], ATLAS [30]	U-Net and CNN hybrid
[23]	128 × 128 (×2)	256 × 256	32.45	0.935	-	ESRGAN
[22]	128 × 128 (×2)	256 × 256	Kirby 21 (37.16), NAMIC (35.56)	Kirby 21 (0.990), NAMIC (0.982)	Kirby 21 [32], NAMIC [33]	ResNet
[19]	128 × 128 × 128 (×2)	256 × 256 × 256	Kirby 21 (38.93), NAMIC (38.06)	Kirby 21 (0.9797), NAMIC (0.9767)	Kirby 21 [32], NAMIC [33]	Deep 3D CNN with skip connections
[18]	93 × 93 × 93 (×2, ×3, ×4)	186 × 186 × 186, 279 × 279 × 279, 372 × 372 × 372	HCP ×2 (35.97), HCP ×3 (32.63), HCP ×4 (30.64)	HCP ×2 (0.9827), HCP ×3 (0.9671), HCP ×4 (0.9519)	Human Connectome Project (HCP) [38]	3D regression-based filters
[17]	40 × 40 (×2)	80 × 80	Kirby 21 (43.68), ANVIL-adult (40.96), MSSEG (41.22)	Kirby 21 (0.9965), ANVIL-adult (0.9906), MSSEG (0.9978)	Kirby 21 [32], ANVIL-adult [39], MSSEG [40]	CNN and ResNet hybrid
[16]	20×20 (×2, ×3, ×4)	40 × 40, 60 × 60, 80 × 80	BrainWeb ×2 (46.58), BrainWeb × 3 (40.97), BrainWeb ×4 (35.20), NAMIC ×2 (38.32), NAMIC ×3 (33.76), NAMIC ×4 (30.84)	BrainWeb ×2 (0.999), BrainWeb ×3 (0.995), BrainWeb ×4 (0.986), NAMIC ×2 (0.945), NAMIC ×3 (0.872), NAMIC ×4 (0.811)	BrainWeb [41], NAMIC [33]	ResNet

## 3. Materials and Methods

**Figure 1:**
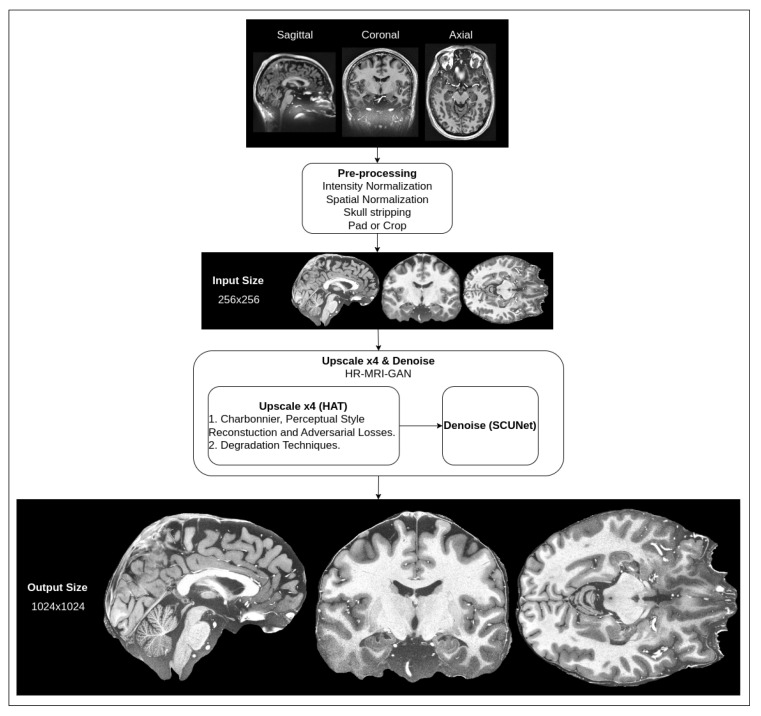
High-level overview of suggested preprocessing pipeline for MRI.

We are proposing a hybrid architecture network that consists of two parts: super-resolution upscaling of MRI slice images and noise removal. As typical MRI images are low-resolution and have natural noise due to the signal being affected by interference and the patient’s body’s radio-frequency emissions [42], it is necessary to apply filtering to reduce noise, as well as to try to improve the resolution of the images while preserving smaller details of the brain. High-resolution MRI images are very expensive to produce because not every hospital has MRI tomographs capable of producing high-resolution images. The proposed methods could be beneficial in clinical practice. A high-level overview of the pipeline for our proposed method for the preprocessing of MRI images is depicted in Figure 1.

**Figure 2:**
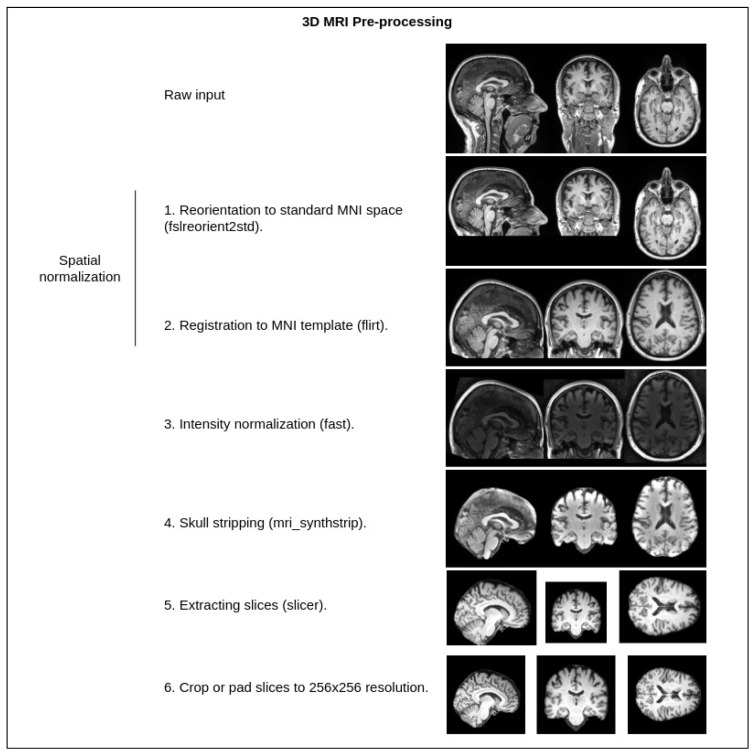
Standard MRI preprocessing steps used in our pipeline. Tools from the FSL [43] library—fslreorient2std, flirt, fast, slicer. Tools from the FreeSurfer [44] library—mri_synthstrip. Crop or pad are implemented with a custom Python script.

The shown pipeline has the default preprocessing steps mentioned (intensity normalization, spatial normalization, skull stripping, pad and crop), which are common for almost every T1w MRI image that is processed in any kind of workflow (the steps are additionally illustrated in Figure 2).

The input for our hybrid network is images that have already been preprocessed with the default steps. In the input, our hybrid network takes a 256 × 256 pixel density resolution MRI slice image, applies four times upscaling with an upscale network and then removes noise with a denoising network. In the output, we get a 1024 × 1024 pixel density image that is filtered of noise. This resolution is approximately equal to a 250 μm in-plane (spatial) resolution.

Our proposed method consists of these parts: an upscale network trained with a combination of pixel/structural-level loss functions and multiple degradation techniques and a denoising network. Each part is detailed in the following sections.

### 3.1. Upscale Network

**Figure 3:**
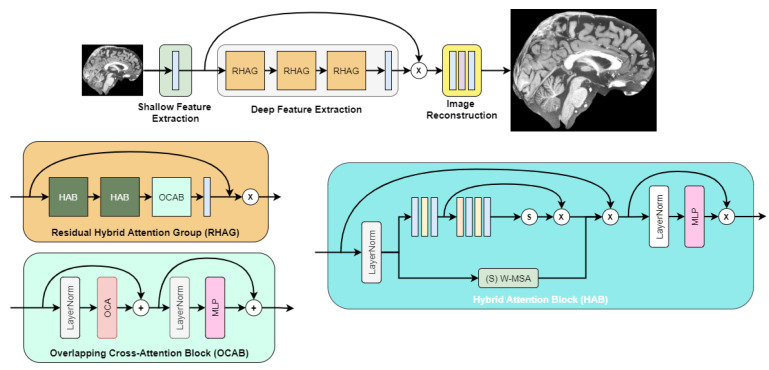
HAT transformer architecture.

After initial experimentation with super-resolution networks, we found that the highest objective quality out of the box was achieved by the image super-resolution transformer called HAT [10]. Its architecture is depicted in Figure 3, as described in the authors’ paper.

**Figure 4:**
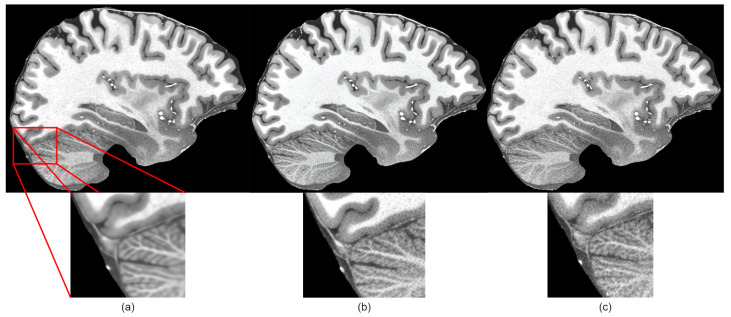
Subjective comparison of sharpness of the proposed method: (**a**) before applying changes to HAT model, (**b**) after applying suggested changes, (**c**) ground truth image.

However, the subjective quality was low because generated images were blurry, as shown in Figure 4a. When compared to other super-resolution networks, we observed that, by improving the HAT transformer with the techniques described in this section, we could make the network generate sharp and closer to ground truth images.

#### 3.1.1. Degradation

Generating realistic images as proposed in recent research [12,13] requires introducing specific degradation techniques for training data. Such techniques include blur, JPEG compression artifacts, artificial noise, cropping, padding, rotating, etc. These degradation techniques are just a combination of different augmentations applied during training for each image from the dataset. These techniques improve the quality of generated images in super-resolution applications when input images might be blurry or noisy.

#### 3.1.2. Loss Functions

To preserve the learning of features at different levels (pixel, structural), we combined a set of loss functions that are designed for respective feature-level learning. Typical choices for pixel-level learning are binary cross-entropy loss, L1 loss and Charbonnier loss [45]. We chose Charbonnier loss as it is a variant of L1 loss that is more stable for outliers. For structural-level and super-resolution learning, we chose to utilize perceptual-style reconstruction loss [46], which has been proven to allow generation of images subjectively close to the ground truth. To further improve the generated images’ quality, we added adversarial loss, which allows the network to generate realistic images.

**Charbonnier loss**. Charbonnier loss is just a differentiable variant of L1 loss (also known as the mean absolute error (MAE)). It has been found [47,48] that this function allows networks to learn more realistic pixel-level features; also, it is a great choice to obtain robustness against overfitting, accuracy and good inference time [49]. Charbonnier loss is defined in Equation (1).

(1) $$L_{Charbonnier}=\frac{\sum_{i=1}^{n}\sqrt{{\left(y_{i}-x_{i}\right)}^{2}+\epsilon^{2}}}{n},$$

**Perceptual-style reconstruction loss**. To allow a network to be able to learn structural features, a common technique is to use perceptual loss functions, which focus on optimizing networks to learn high-level-style features. One such loss function is perceptual-style reconstruction loss, first proposed in [50,51]. The main idea for this loss function is to take a deep convolutional neural network (VGG-19 was originally used [52]) pretrained on a large dataset, like ImageNet [53], and extract activations from deep layers that have learned high-level features from the dataset. These features are held in common between the majority of objects and can represent the semantics of the images. The loss function yields higher error values if the generated image differs in texture, colors, brightness, etc. The mathematical expression of these proposed ideas is represented by the *Gram* matrix, which is defined in Equation (2).

(2) $$G_{j}^{\phi }{\left(x\right)}_{c,c^{{}^{\prime }}}=\frac{1}{C_{j}H_{j}W_{j}}\sum_{h=1}^{H_{j}}\sum_{w=1}^{W_{j}}\phi_{j}{\left(x\right)}_{h,w,c}\phi {\left(x\right)}_{h,w,c^{{}^{\prime }}},$$

where $\phi_{j}\left(x\right)$ are activations of image *x* in convolution layer *j* of shape $C_{j}$×$H_{j}$×$W_{j}$.

Reshaping this Gram matrix into matrix ψ with shape $C_{j}$×$H_{j}W_{j}$, we get Equation (3), which makes it possible to calculate the matrix efficiently. With a reshaped Gram matrix ψ, we can define perceptual-style reconstruction loss as in Equation (4).

(3) $$G_{j}^{\phi }\left(x\right)=\frac{\psi \psi^{T}}{C_{j}H_{j}W_{j}},$$

(4) $$L_{style}^{\phi ,j}\left(\hat{y},y\right)={∥G_{j}^{\phi }\left(\hat{y}\right)-G_{j}^{\phi }\left(y\right)∥}_{F}^{2},$$

where $\hat{y}$ is the generated image and *y* is the target (ground truth) image. The application of this loss function in training allows a network to generate perceptually and semantically similar images to the ones used in training. One examples is given in [46], which shows the style transfer effect from one image to another.

**Adversarial Loss**. Another improvement that allows networks to generate hyper-realistic images is to take advantage of adversarial loss, first proposed in [15]. This type of loss (shown in Equation (5)) introduces generative adversarial networks (GANs), which make use of an architecture that has two networks: a generator and discriminator. The generator only focuses on generating an image that cannot be distinguished as a fake from the real, and the discriminator’s objective is to correctly distinguish fake from real images. We used binary cross-entropy loss for both the generator and discriminator because we wanted the discriminator to assign high probability (close to 1) for generator outputs and the generator to generate images from an input feature vector that is a low-resolution image instead of a random latent vector *z* like in the original proposed GANs. The objective of the generator is to minimize the adversarial loss, while discriminators aim to maximize it. The generator in our case was the HAT model and the discriminator was the U-Net model described in [12] with a minor modification: instead of the default number of output channels in convolution layers (64), we used 128. The adversarial BCE loss function we used is defined in Equation (6). To define the objective loss functions for the generator and discriminator, we replace the terms in Equation (5) with the BCE loss function. For the generator, we get the loss function as defined in Equation (7), and for the discriminator, we get the loss function as defined in Equation (8):

(5) $$\min_{G}\max_{D}V\left(D,G\right)=\left[\log D(x)\right]+\left[\log\left(1-D(G(z))\right)\right],$$

where *x* is an image, *z* is a random noise latent vector, *D* is the discriminator network output and *G* is the generator network output;

(6) $$L_{BCE}\left(x,y\right)=-\left[y\log\sigma (x)+(1-y)*\log1-\sigma (x)\right],$$

where σ is a sigmoid activation function and *y* is the predicted label (real, fake);

(7) $$L_{G}=L_{BCE}\left(1,D\left(G\left(z\right)\right)\right),$$

where *z* is a fake image;

(8) $$L_{D}=L_{BCE}\left(1,D\left(x\right)\right)+L_{BCE}\left(1,1-D\left(G\left(z\right)\right)\right),$$

where *x* is a real image and *z* is a fake image.

**Combined loss function**. To train the HAT generator, we combined Charbonnier loss, perceptual loss and generator adversarial loss, as defined in Equation (9). To train the U-Net discriminator, we used discriminator adversarial loss.

(9) $$L_{COMB}=L_{Charbonnier}+L_{style}+L_{G}.$$

### 3.2. Denoise Network

To reduce noise in upscaled MRI images, we objectively and subjectively evaluated multiple denoise filters (non-local means [24], anisotropic diffusion [25], the bilateral filter [26], the Chambolle filter [54], the Bregman filter [55], the wavelet filter [56], the median filter [57] and the Gaussian filter [57]), as well as GAN-based solutions (SCUNet [58], SwinIR [11], Restormer [59], PNGAN [60] and NAFNet [61]). The implementation of the non-local means filter from OpenCV [62]; the implementations of the bilateral, Chambolle, Bregman and wavelet filters from the Python library scikit-image [63]; and the median and Gaussian filter implementations from the Python library scipy [64] were used. The performance of each network and filter was evaluated separately (each network and filter was applied independently to the validation dataset, which was used by the upscale network, and results are reported in Table 2).

**Figure 5:**
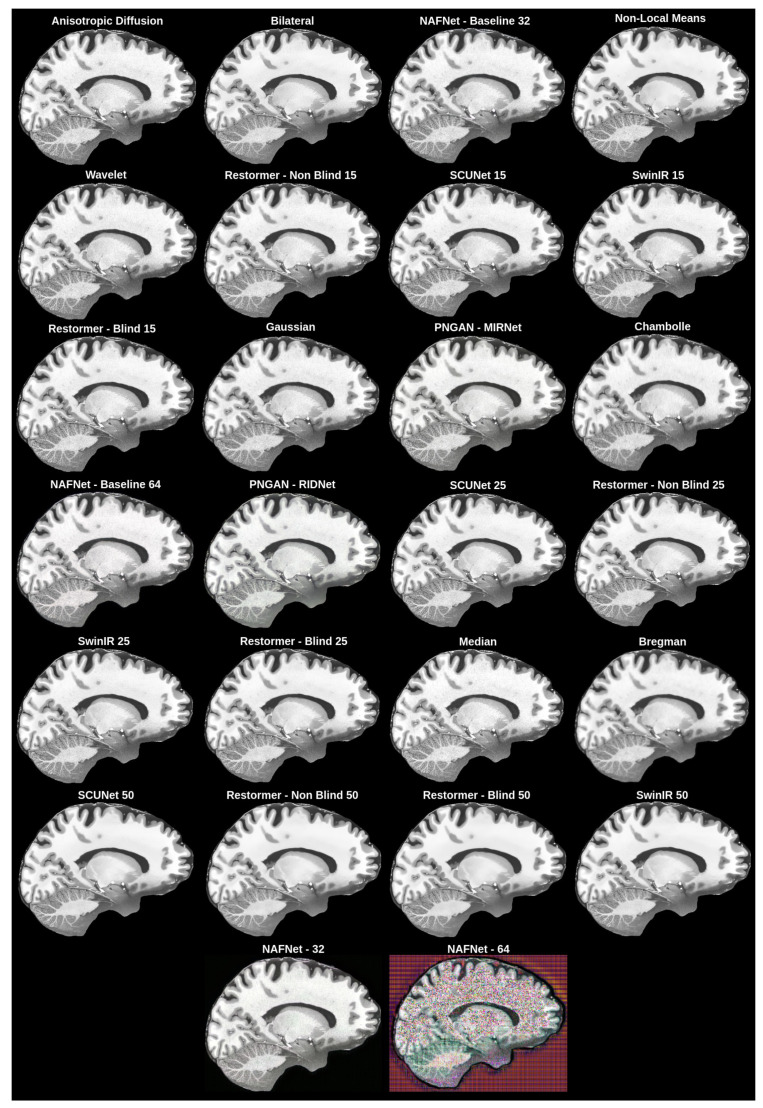
Subjective comparison of denoising filters and models.

The best overall solution, which matched our subjective expectations and had good enough values for the objective evaluation metrics (Section 3.3.1), was chosen as the denoising network method. The objective evaluation is represented in Table 2, while the subjective evaluation is shown in Figure 5.

**Figure 6:**
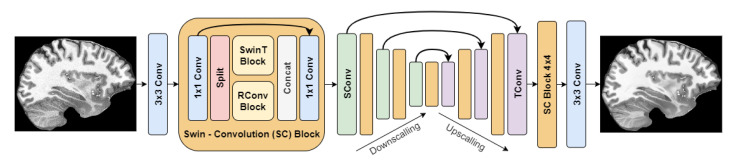
SCUNet architecture.

The best method based on the objective and subjective evaluations was SCUNet with noise level reduction σ = 25. Its architecture is depicted in Figure 6.

### 3.3. Evaluation of Results

Evaluation of the quality of generated images can be undertaken in two ways: objectively and subjectively.

#### 3.3.1. Objective Evaluation

For objective evaluation, there are a couple of commonly used metrics: the peak-signal-to-noise ratio (PSNR) and structural similarity index measure (SSIM) [65]. However, these metrics only capture pixel-level quality, they do not capture the perceptual quality of the image. For that, typical metrics are VSI or LPIPS. As suggested in [66], for MR images, VSI is one of the top-performing quality metrics.

**Peak-signal-to-noise ratio**. The PSNR measures the ratio between the highest possible pixel value (255) and the pixel intensity differences. The metric is expressed in logarithmic decibels and makes it possible to measure how well an image is enhanced compared to the baseline. Higher metric values indicate better image quality. However, this metric only captures pixel-level differences, so if an image that is completely blurry is being compared to a non-blurry one, the metric will yield a high score anyway, even though the perceptual quality of the image is poor. The PSNR metric is defined in Equation (10).

(10) $$PSNR=10\log_{10}\left(\frac{255^{2}}{MSE}\right),$$

where *MSE* is the mean squared error or L2 loss defined in Equation (11).

(11) $$MSE=\frac{1}{m*n}\sum_{i=0}^{m-1}\sum_{j=0}^{n-1}{\left[I(i,j)-K(i,j)\right]}^{2},$$

where an *m* × *n* sized image *I* is approximated by image *K*, and *i, j* are counters for each image dimension.

**Structural similarity index measure**. The SSIM metric is another perceptual metric that allows objectively measuring difference between two images. “Structural” in the metric name indicates that the metric value depends on the visible structure distortions in the image. More distortions degrade the quality of an image and lower the metric value. The metric consists of three parts: luminance, contrast and structure. The general equation for the SSIM is defined in Equation (12), the luminance term in Equation (13), the contrast term in Equation (14) and the structure term in Equation (15).

(12) SSIM(x,y)=l(x,y)c(x,y)s(x,y),

(13) $$l\left(x,y\right)=\frac{2\mu_{x}\mu_{y}+C_{1}}{\mu_{x}^{2}+\mu_{y}^{2}+C_{1}},$$

(14) $$c\left(x,y\right)=\frac{2\sigma_{x}\sigma_{y}+C_{2}}{\sigma_{x}^{2}+\sigma_{y}^{2}+C_{2}},$$

(15) $$s\left(x,y\right)=\frac{\sigma_{xy}+C_{3}}{\sigma_{x}\sigma_{y}+C_{3}},$$

where μ is the mean, σ is the standard deviation and $\sigma_{xy}$ is the cross-covariance of images *x* and *y*.

**Visual saliency-induced index**. The VSI metric [67] is a metric that is oriented to capturing the perceptual quality of an image. The Kadid-10k IQA (image quality assessment) database [68], which was created to evaluate metrics that capture perceptual image quality, has proven that the VSI metric is one of the best-performing when assessing perceptual image quality. The metric first transforms RGB images into other color spaces with a transformation matrix, as shown in Equation (16).

(16) $$\left[\begin{array}{c} L\\ M\\ N \end{array}\right]=\left[\begin{array}{ccc} 0.06 & 0.63 & 0.27\\ 0.30 & 0.04 & -0.35\\ 0.34 & -0.6 & 0.17 \end{array}\right]\left[\begin{array}{c} R\\ G\\ B \end{array}\right]$$

Then, additionally, authors have mentioned that they computed the gradient modulus (GM)—or, in other words, the image gradient—with a Scharr gradient operator. Partial derivatives for the image are calculated as in Equations (17) and (18).

(17) $$G_{x}\left(x\right)=\frac{1}{16}\left[\begin{array}{ccc} 3 & 0 & -3\\ 10 & 0 & -10\\ 3 & 0 & -3 \end{array}\right]*f\left(x\right),$$

(18) $$G_{y}\left(x\right)=\frac{1}{16}\left[\begin{array}{ccc} 3 & 10 & 3\\ 0 & 0 & 0\\ -3 & -10 & -3 \end{array}\right]*f\left(x\right),$$

Then, the GM is computed as in Equation (19).

(19) $$G\left(x\right)=\sqrt{G_{x}^{2}\left(x\right)+G_{y}^{2}},$$

The last part, which is also captured additionally to the GM, is the visual saliency (VS) map, which is extracted with a trained model for each image. These maps are then used to calculate similarity between different image features (VS maps, GM and chrominance elements). Similarity between VS maps is calculated as in Equation (20).

(20) $$S_{VS}\left(x\right)=\frac{2VS_{1}\left(x\right)\cdot VS_{2}\left(x\right)+C_{1}}{VS_{1}^{2}\left(x\right)+VS_{2}^{2}\left(x\right)+C_{1}},$$

where $C_{1}$ is a constant that controls the stability of the similarity between VS maps. Similarity between GMs for images is then computed, as denoted in Equation (21).

(21) $$S_{G}\left(x\right)=\frac{2G_{1}\left(x\right)\cdot G_{2}\left(x\right)+C_{2}}{G_{1}^{2}\left(x\right)+G_{2}^{2}\left(x\right)+C_{2}},$$

where $C_{2}$ is another constant but this it time controls the stability for the GM similarity. One of the last parts in the VSI metric is the chrominance element. Chrominance is captured after RGB image transformation, where we get *L, M, N* channels. Then, the chrominance similarity is calculated as in Equation (22).

(22) $$S_{C}\left(x\right)=\frac{2M_{1}\left(x\right)\cdot M_{2}\left(x\right)+C_{3}}{M_{1}^{2}\left(x\right)+M_{2}^{2}\left(x\right)+C_{3}}\cdot \frac{2N_{1}\left(x\right)\cdot N_{2}\left(x\right)+C_{3}}{N_{1}^{2}\left(x\right)+N_{2}^{2}\left(x\right)+C_{3}},$$

where $C_{3}$ is a positive constant as well. Combining the captured similarities, we get the similarity measure denoted in Equation (23).

(23) $$S\left(x\right)=S_{VS}\left(x\right)\cdot {\left[S_{G}\left(x\right)\right]}^{\alpha }\cdot {\left[S_{C}\left(x\right)\right]}^{\beta },$$

where α and β are controllable parameters that control the importance of the GM and chrominance components. Finally, the VSI metric equation is provided in Equation (24).

(24) $$VSI=\frac{\sum_{x\in \Omega }S\left(x\right)\cdot VS_{m}\left(x\right)}{\sum_{x\in \Omega }VS_{m}\left(x\right)},$$

where S(x) is the local similarity of image $f_{1}$ and image $f_{2}$, $VS_{m}$ is the *max* value between $VS_{1}\left(x\right)$ and $VS_{2}\left(x\right)$ and Ω denotes the whole spatial domain.

**Learned perceptual image patch similarity**. The LPIPS metric was first introduced in [69] and is an extension of the perceptual-style reconstruction loss but as a metric. This metric also extracts features from deep layers and computes distances between them. The authors of the metric mentioned that it is capable of representing human perceptual similarity judgment well and can be used as an objective evaluation metric to capture the subjective component. The metric was also mentioned in the Kadid-10k benchmark as one of the best perceptual image quality evaluation metrics, together with the VSI metric.

#### 3.3.2. Subjective Evaluation

Every person understands what good quality is differently. In the case of super-resolution upscaling and denoising of images, subjective evaluation is not difficult because generated images may contain differences, distortions, blurriness and noise that differ from the ground truth. An image with distortions is of poor quality. Even though the VSI and LPIPS metrics have proven that objective evaluation can capture the subjective component, it is still preferred to have humans included in the evaluation loop to make the final decision.

## 4. Results and Discussion

### 4.1. Experimentation Data

For the experiment, we utilized the ultra-high-resolution MRI dataset “human phantom” [70] with isotropic resolution of 250 μ for T1w MRI scans (dataset available online: https://datadryad.org/stash/dataset/doi:10.5061/dryad.38s74, accessed on 31 August 2023). In Table 3, we provide a list of studies where this dataset has been used or mentioned.

**Figure 7:**
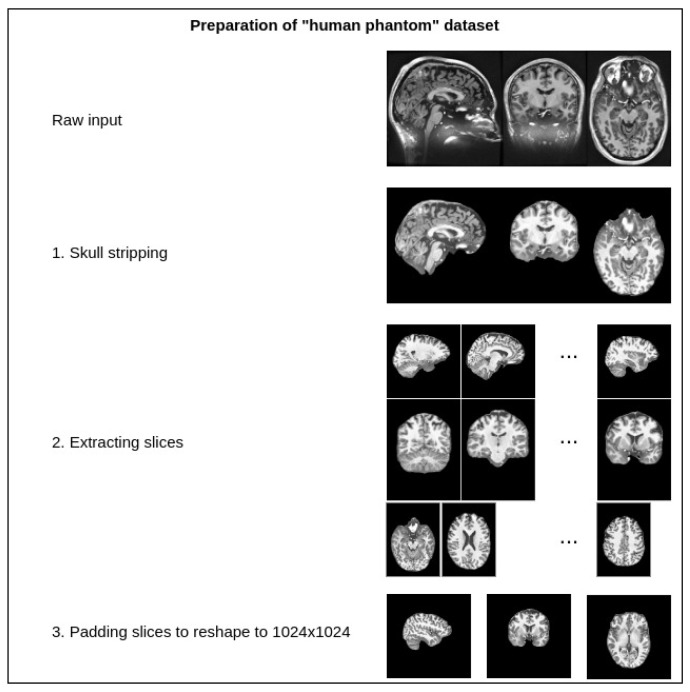
Preparation steps for the human phantom dataset.

The dataset contains one intensity-normalized and spatially normalized T1w MRI scan of a patient. The shape of this scan is 640, 880, 880. We performed skull stripping on the mentioned scan and then extracted the slices for all planes, removed empty slices or slices without enough relevant information and combined them into one dataset. Finally, all extracted slice images were padded with zeros to give them a square shape of 1024 × 1024. These steps are illustrated in Figure 7. This dataset was then split into training and validation sets with a random sampling in a ratio of 80 to 20 percent. Sampled projections from slices were kept together; for example, for slice number 171, we moved all three planes into either the training or validation set. The sampling was undertaken in terms of the slices and not the extracted projections. The final pixel resolution for ground truth images was 1024 × 1024. To acquire low-resolution images, we applied bicubic downsampling using the Python package Pillow and reduced the pixel resolution to 256 × 256.

To verify the generalizability of the created model, we utilized a test set from OASIS 4 [76] that consists of different T1w scans for patients with dementia. In the dataset, the scans are from different MR tomographs with different Tesla configurations. All plane slices of scans after default preprocessing had 256 × 256 pixel resolution.

### 4.2. Implementation Details

For the training environment, we used a personal computer with an AMD Ryzen 5900 X CPU, GeForce RTX 3090 GPU and 32GB RAM. For the final model, we used a batch size of 4, a patch size of 64 × 64 and an Adam optimizer with a learning rate of 0.0001, which was gradually decreased by 0.5 at 50,000, 125,000, 200,000, 225,000 and 240,000 steps. The HAT generator model architecture was not modified. As mentioned previously, for the discriminator, we used the U-Net model proposed in [12] with 128 output channels in convolution layers instead of 64.

### 4.3. Results

The creation of the proposed methodology started with training many different models for the super-resolution problem. As mentioned before, we trained the HAT model in the default configuration, as proposed by previous authors [10] and in work on CARN [14], BSRGAN [13], SwinIR [11] and Real-ESRGAN [17]. Then, we compared the results that we got for all models with our validation set objectively and subjectively. Objective evaluation is shown in Table 4.

**Figure 8:**
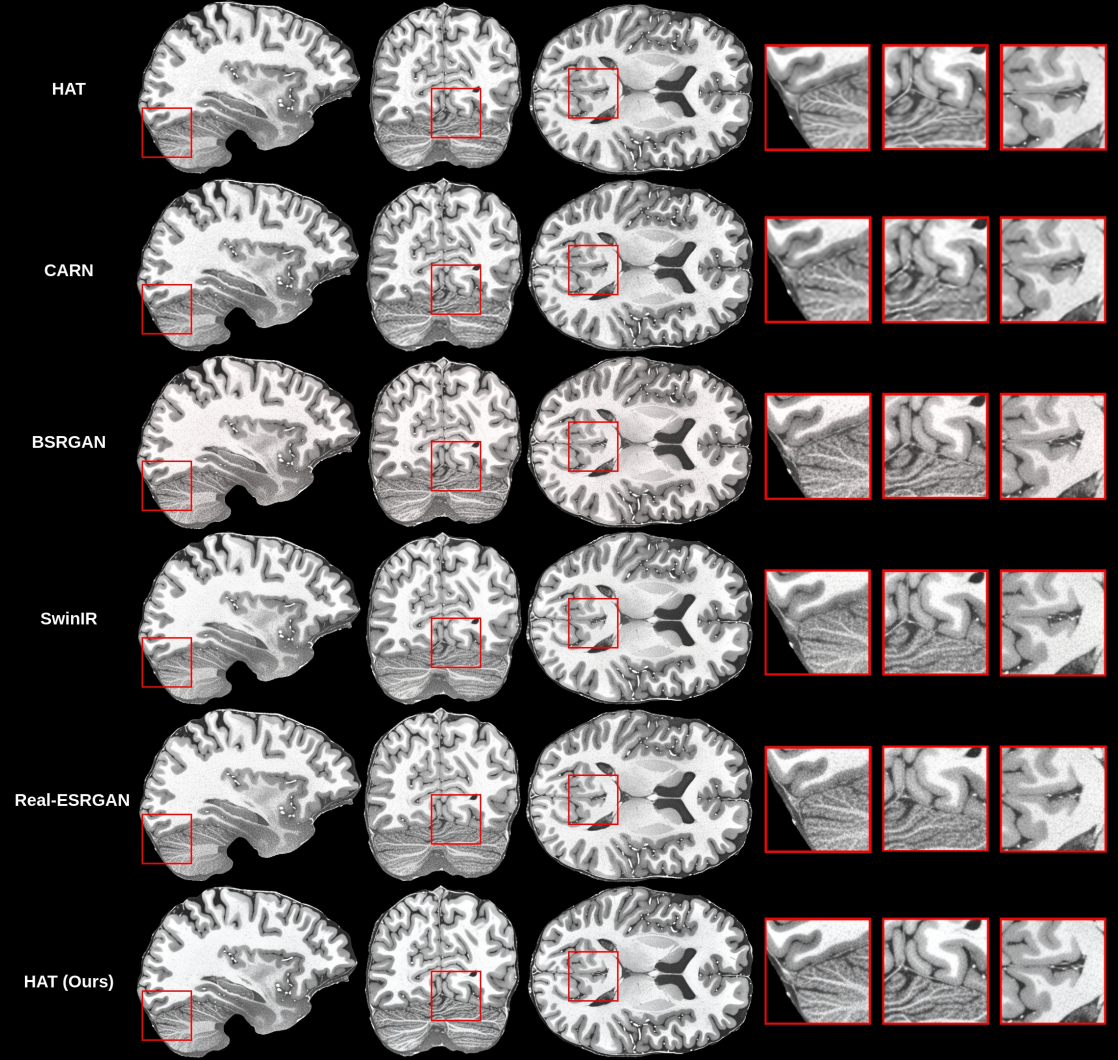
Subjective comparison of upscale models.

Initial findings showed that the HAT model in the default configuration was capable of upscaling MRI while preserving good quality because the SSIM and PSNR were the highest among the trained models, but visually the images were blurry. This can be seen in the subjective comparison in Figure 8. Both the default HAT and CARN models produced blurry results because models with default settings do not use perceptual-style loss or the adversarial training technique. Looking both at the metric results and the visual quality of the upscaled images, we decided to improve the HAT model since it produced the best overall results by applying the proposed methodology.

**Figure 9:**
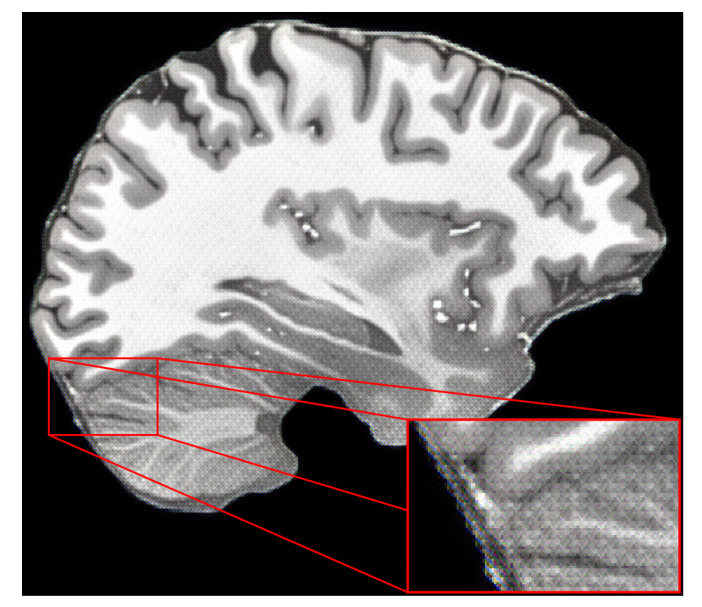
Results of using only perceptual-style reconstruction loss with Charbonnier loss for training the HAT model.

The first change was to include the perceptual-style reconstruction loss in the training pipeline together with Chambonnier loss. However, the results were not satisfactory because upscaled images had artifacts, as shown in Figure 9. The findings showed that perceptual-style reconstruction loss alone was not enough to preserve sharpness while upscaling the MR images. The next step was to include the adversarial loss, as defined in our methodology.

After adversarial loss inclusion, we found that it dramatically improved the sharpness while increasing the resolution of MR images. This can be seen in Figure 8. Next, we introduced the VSI and LPIPS perceptual quality metrics into our considerations. We calculated both metrics for each trained model and report the results in addition to the SSIM and PSNR metrics. In Table 4, we can see that the VSI metric had the highest score for the default HAT model, even though the visual quality was not the highest because the upscaled images were blurry. This finding was not what we expected from a perceptual quality metric. However, the LPIPS metric did match our perceptual judgment of upscaled images and showed objectively that our improved HAT model outperformed other state-of-the-art models in terms of perceptual image quality.

The second step in our proposed methodology is MRI denoising. We took a number of widely known image filters and a couple of SOTA denoising networks and applied them to evaluate objectively and subjectively which filter or network would work the best in terms of the MRI denoising problem. The objective evaluation is shown in Table 2. We calculated the same metrics for all filters, networks and their modifications. All the networks that we evaluated used open-source-community shared weights.

The best objective results were achieved with the anisotropic diffusion filter; however, it was impossible to see if any noise was removed from the images, as shown in Figure 5.

To choose the best overall network for the MRI denoising problem, we subjectively evaluated different denoising filters and models. Our initial intention was to obtain a filter or a network that could remove the noise from MRI. As a result, as can be seen in Figure 5, we found that it was impossible to remove all noise because, as filters became more aggressive, more distortions of the ground truth appear. We can even see failed tries, such as NAFNet with a filter width of 64, where upscaled images had strange pixel artifacts. This happened due to the network being unable to cope with the MRI dataset we used.

For the denoising method, we chose the middle ground between the results with the most noise and the least noise, which had high perceptual quality and good metric results. The chosen network was SCUNet with a noise reduction level of 25, as mentioned in the methodology.

**Figure 10:**
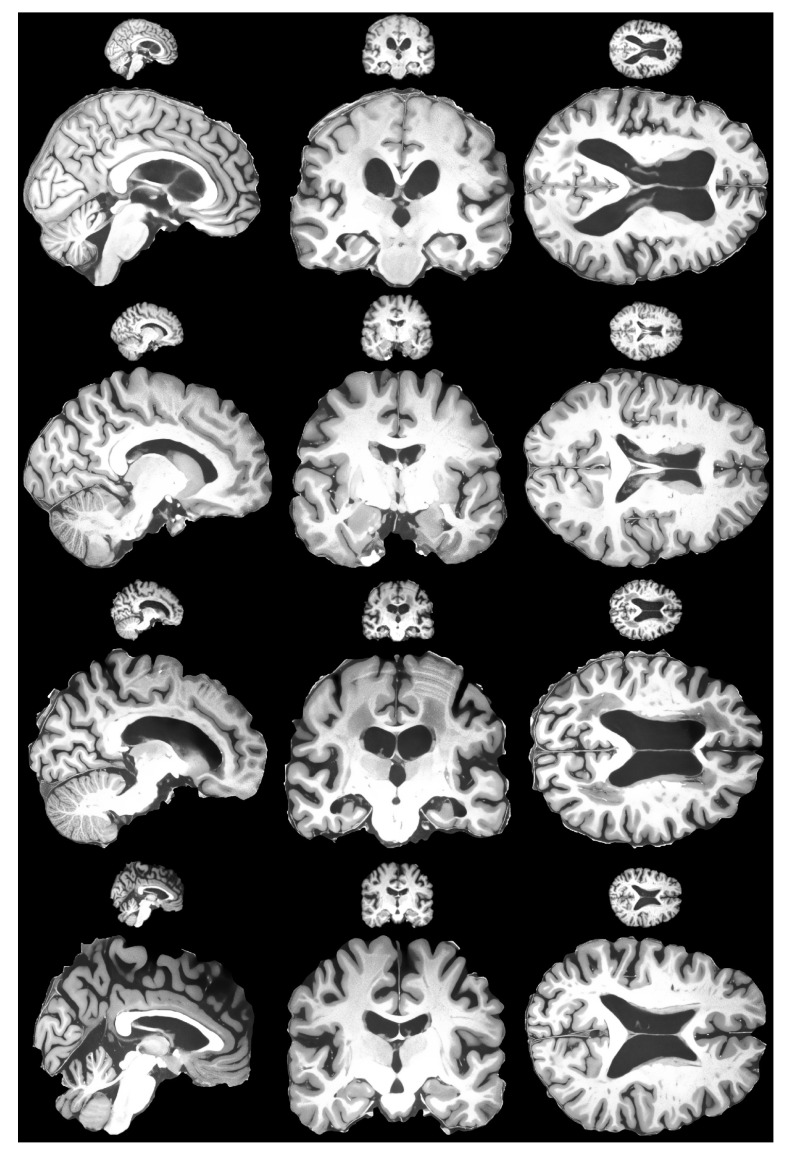
Subjective comparison of HR-MRI-GAN model results with OASIS 4 dataset. Figure shows four different patients’ brain scan slices for each plane.

The last step was to confirm that the proposed HR-MRI-GAN pipeline works well with unseen data. We tested the model with the OASIS 4 dataset [76]. Since the OASIS 4 dataset ground truth images have 256 × 256 resolution, we could not objectively evaluate the results. However, we could evaluate subjectively. The results are depicted in Figure 10. Judging the results, it is fair to say that our model generalized well with unseen data due to the degradation techniques applied during training, as proposed by the defined methodology, and the generated images were of high perceptual quality. The goal of preserving small details and removing as much noise as possible was achieved successfully.

### 4.4. Discussion

A special problem in picture enhancement activities is managing the trade-off between pixel-level quality and perceived quality. Previous techniques have concentrated largely on maximizing one component without thoroughly investigating the trade-off. The strategies adopted constitute a happy medium between high pixel-level similarity and perceived quality. These strategies were most likely chosen based on the experimental findings, which revealed a trade-off between pixel-level and perceived quality. To achieve more stable training and better optimum solutions, the suggested modifications include employing a denser and deeper network (VGG-16) for the discriminator, boosting the self-attention layers in the HAT model and using a Wasserstein GAN (WGAN). The hybrid combination is a revolutionary method for medical image enhancement that capitalizes on the strengths of both designs for superior results. The results of this paper demonstrate that the proposed hybrid transformer generative adversarial network (HT-GAN) method for improving the perceptual quality of MR images through joint denoising and super-resolution upscaling outperforms state-of-the-art methods in terms of both quantitative and qualitative evaluation metrics. We conducted extensive experiments with an ultra-high-resolution MRI dataset and a publicly available sMRI dataset, and the results showed that the proposed method significantly improved the accuracy of subsequent analysis and diagnosis of sMRI images. The proposed methodology has the potential to be applied in clinical practice and can significantly improve the quality of medical images, ultimately leading to better patient outcomes. The results of this paper demonstrate the potential of deep learning techniques, specifically GANs and transformers, in improving the quality of medical images and enhancing the accuracy of subsequent analysis and diagnosis.

The proposed methodology is a middle ground between high pixel-level similarity and perceptual image quality based on our experimental results. Based on the comparison of the metrics in Table 4, we can see that the pixel-level quality (SSIM and PSNR metrics) was reduced after applying perceptual quality-preserving techniques. In some cases, we can see that very small details were washed out or missing in the upscaled images, like in the example in Figure 4. However, upscaled images were no longer blurry and better in terms of perceptual quality. In the future, it would be beneficial to try to preserve better pixel-level quality. At least a couple of improvements can be made to achieve this. The first improvement would be changing the discriminator network to a denser and deeper network, like VGG-16, which would be far more capable of capturing small details in images since these networks are known in the field to be great image classifier backbones. For the second improvement, we could increase the number of self-attention layers in the HAT model, which may additionally improve the quality of upscaled images. This is one of the techniques that the authors of the HAT model used to improve metric results with the HAT-L modification, which had two times more attention layers. For the third improvement, we could use the Wasserstein GAN (WGAN) [77], which is a modification of the GAN proposed by [15]. The WGAN has been proved to be more stable during training and could help in finding better optimal solutions, which would be equivalent to higher-quality upscaled images. We are planning to apply these additional improvements in the future.

Another aspect that is worth discussing is why the VSI metric did not reflect our subjective evaluation of upscaled images as well as the LPIPS. Our expectations were high, since the Kadid-10k benchmark results proposed it as the best-performing metric in terms of perceptual image quality. We believe, and our results have shown, that the metric itself is not suitable for the problem we are addressing: grayscale image perceptual quality evaluation. The metric was developed for RGB images and not for single-channel images. Even if we use the basic approach of cloning one channel to the other two when converting a grayscale image to an RGB one, the metric still does not work as expected. It is evident that the VSI metric is not suitable for MRI perceptual quality evaluation. For grayscale image perceptual quality evaluation, the LPIPS metric is a better choice.

The application of SCUNet for the MRI denoising problem was successful. Even though the model weights were not specifically trained for MRI, the result was still satisfactory enough. This means that general-purpose grayscale denoising networks can be applied to the MRI denoising problem. Since MR images naturally come with noise, it is impossible to find a dataset for which MR images would be noise-free. In the future, it would be beneficial to work on a solution that would allow application of transfer learning from general-purpose denoising networks to MRI denoising. This could potentially improve the final image quality even more.

Recently, ensemble learning has been becoming a popular topic in the research community. From the machine learning perspective, an ensemble is a collection of models trained to solve the same problem but using different model types or data. Typically, ensembles tend to increase the performance of traditional models due to the statistically increased probability of achieving a better model when training multiple different models for an ensemble [78]. Ensemble learning could also be used for the super-resolution task, where multiple different super-resolution models could be trained and then the results of the model with the best metric results (or the median) would be used as an output. In the same way, outputs from all models could be averaged to a single output. There are plenty of examples where ensemble learning improves network performance: predicting the functional brain connectome [79], detection of Alzheimer’s disease [80], flood prediction [81] and change point estimation [82].

## 5. Conclusions

In this paper, we introduced an innovative approach aimed at enhancing the perceptual quality of MR images through the utilization of a hybrid transformer generative adversarial network (HT-GAN). By synergistically leveraging the capabilities of both generative adversarial networks (GANs) and transformers, our proposed method presents a unified solution to jointly address the challenges of denoising and the super-resolution task within the realm of structural MRI (sMRI) enhancement.

Our contributions extend to modifying the hybrid attention transformer (HAT) model to heighten the perceptual image quality of MRI. Demonstrating superiority over existing state-of-the-art (SOTA) super-resolution networks, our method, aptly named HR-MRI-GAN, exhibits remarkable perceptual image quality enhancement. Furthermore, the versatility of our approach is evident in its ability to generalize effectively to previously unseen data. Seeking to further elevate MRI quality, we incorporated advanced denoising networks, showcasing the adaptability of general-purpose SOTA denoising models to the intricate domain of MRI denoising. Notably, our exploration uncovered the limitations of the visual saliency-induced index (VSI) metric for evaluating MRI perceptual image quality, steering us toward more appropriate evaluation criteria.

The empirical outcomes of our study underscore the remarkable efficacy of the proposed HR-MRI-GAN method, surpassing prevailing benchmarks in both quantitative and qualitative evaluation domains. Beyond the realms of research, the proposed methodology carries implications of considerable practical significance, potentially revolutionizing the accuracy of subsequent sMRI image analysis and diagnostic procedures. As we navigate towards the prospect of clinical implementation, this work paves the way for substantial improvements in medical image quality and, by extension, patient care. The synergistic fusion of GANs and transformers offers a promising avenue for propelling the field of medical imaging forward, underscoring the tremendous potential of deep learning techniques in transforming healthcare practices.

The intricacy and computational demands of combining GANs and transformers may result in lengthier training timeframes and resource-intensive processing as compared to more standard techniques. Furthermore, the trade-off between pixel-level and perceived quality may result in fine detail loss in upscaled pictures. In future work, we plan to explore the optimization of these aspects, as well as the potential of transfer learning from general-purpose denoising networks to MRI denoising, which could potentially improve the final image quality even more. Additionally, we plan to investigate the use of our proposed methodology on other types of medical images, such as CT scans and PET scans. Overall, our proposed methodology has the potential to significantly improve the quality of medical images and enhance the accuracy of subsequent analysis and diagnosis, ultimately leading to better patient outcomes.

## Figures and Tables

**Table 2:** Comparison of evaluated denoise models and methods.

Model	Modification	SSIM (%) ↑	PSNR (dB) ↑	VSI ↑	LPIPS ↓
Anisotropic diffusion	Kappa = 60, gamma = 0.0135	99.57	45.07	0.9992	0.0048
Bilateral filter	σ(5)	98.55	39.31	0.9943	0.0209
NAFNet	Baseline and width 32	97.85	36.55	0.9978	0.0305
Non-local means	σ(10)	96.25	38.44	0.9958	0.0487
Wavelet filter	Wavelet = “sym9”	96.22	34.72	0.9984	0.0631
Restormer	Non-blind and σ(15)	96.19	35.34	0.9970	0.0349
SCUNet	σ(15)	96.18	35.35	0.9964	0.0348
SwinIR	σ(15)	96.12	35.36	0.9965	0.0376
Restormer	Nlind and σ(15)	96.09	35.31	0.9966	0.0364
Gaussian filter	Std = 0.75	95.96	34.14	0.9979	0.0489
PNGAN	MIRNet	95.81	35.23	0.9974	0.0587
Chanbolle filter	Weight = 0.08	95.11	34.84	0.9969	0.0886
NAFNet	Baseline and width 64	94.87	34.38	0.9976	0.1555
PNGAN	RIDNet	94.08	34.09	0.9971	0.0833
**SCUNet**	σ(25)	**93.94**	**33.29**	**0.9949**	**0.0553**
Restormer	Non-blind and σ(25)	93.85	33.24	0.9949	0.0580
SwinIR	σ(25)	93.79	33.28	0.9947	0.0636
Restormer	Blind and σ(25)	93.78	33.24	0.9947	0.0577
Median filter	Kernel size = 2	93.71	30.12	0.9947	0.0384
Bregman filter	Weight = 4.5	91.46	32.02	0.9949	0.0991
SCUNet	σ(50)	91.07	31.19	0.9903	0.0903
Restormer	Non-blind and σ(50)	90.76	31.09	0.9899	0.0995
Restormer	Blind and σ(50)	90.68	31.09	0.9898	0.1013
SwinIR	σ(50)	90.38	31.13	0.9904	0.1157
NAFNet	Width 32	17.83	21.38	0.9796	0.5353
NAFNet	Width 64	16.78	15.00	0.9349	0.5685

σ—Noise level reduction factor, SCUNet—chosen network for MRI denoising.

**Table 3:** References to the “human phantom” dataset in other studies.

Reference	Description
[71]	Literature review on how high-resolution MRI can help in the detection of amyotrophic lateral sclerosis. The dataset was used to justify how certain vascular markers can be identified in the brain due to high-resolution MRI making it possible to see small details, which can be crucial for detection of some diseases, including amyotrophic lateral sclerosis.
[72]	Book chapter where usage of high-resolution MRI is discussed—how small details in brain imaging can help in assessment of neurodegenerative pathophysiology and vascular dysfunction. The dataset was mentioned as an example.
[73]	The research utilized the dataset in quantitative susceptibility mapping (QSM) MRI reconstruction from thin slices, where a T1w scan was used as a structural reference. This research aimed to improve QSM reconstruction speed and reliability.
[74]	Literature review conducted to analyze the current state of ultra-high-resolution MRI acquisition in Germany. The dataset was mentioned as one of the sources for high-resolution MRI.
[75]	Book chapter that discusses state-of-the-art methods and datasets for ultra-high-resolution structural MRI acquisition. The dataset was mentioned as an example.

**Table 4:** Comparison of upscale models’ validation metric results.

Model	SSIM ↑	PSNR ↑	VSI ↑	LPIPS ↓
HAT [10]	**91.40**	**31.76**	**0.9971**	0.0984
CARN [14]	90.70	30.43	0.9963	0.0964
HAT (ours)	88.58	28.74	0.9942	**0.0529**
BSRGAN [13]	87.96	28.42	0.9944	0.0542
SwinIR [11]	87.76	28.25	0.9937	0.0546
Real-ESRGAN [12]	86.96	27.24	0.9915	0.0585

## Data Availability

Not applicable.

## References

[1] Krishnapriya S., Karuna Y. (2023). A survey of deep learning for MRI brain tumor segmentation methods: Trends, challenges, and future directions. Health Technol..

[2] Khan S.U., Ullah N., Ahmed I., Ahmad I., Mahsud M.I. (2019). MRI imaging, comparison of MRI with other modalities, noise in MRI images and machine learning techniques for noise removal: A review. Curr. Med Imaging.

[3] Odusami M., Maskeliūnas R., Damaševičius R. (2023). Pixel-Level Fusion Approach with Vision Transformer for Early Detection of Alzheimer’s Disease. Electronics.

[4] Praveen S.P., Srinivasu P.N., Shafi J., Wozniak M., Ijaz M.F. (2022). ResNet-32 and FastAI for diagnoses of ductal carcinoma from 2D tissue slides. Sci. Rep..

[5] Ullah I., Ali F., Shah B., El-Sappagh S., Abuhmed T., Park S.H. (2023). A deep learning based dual encoder–decoder framework for anatomical structure segmentation in chest X-ray images. Sci. Rep..

[6] Dong C., Loy C.C., He K., Tang X. (2015). Image Super-Resolution Using Deep Convolutional Networks. arXiv.

[7] Vaswani A., Shazeer N., Parmar N., Uszkoreit J., Jones L., Gomez A.N., Kaiser L., Polosukhin I. (2017). Attention Is All You Need. arXiv.

[8] Cordonnier J.B., Loukas A., Jaggi M. (2019). On the Relationship between Self-Attention and Convolutional Layers. arXiv.

[9] Dosovitskiy A., Beyer L., Kolesnikov A., Weissenborn D., Zhai X., Unterthiner T., Dehghani M., Minderer M., Heigold G., Gelly S. (2020). An Image is Worth 16x16 Words: Transformers for Image Recognition at Scale. arXiv.

[10] Chen X., Wang X., Zhou J., Dong C. (2022). Activating More Pixels in Image Super-Resolution Transformer. arXiv.

[11] Liang J., Cao J., Sun G., Zhang K., Van Gool L., Timofte R. (2021). SwinIR: Image Restoration Using Swin Transformer. arXiv.

[12] Wang X., Xie L., Dong C., Shan Y. (2021). Real-ESRGAN: Training Real-World Blind Super-Resolution with Pure Synthetic Data. arXiv.

[13] Zhang K., Liang J., Van Gool L., Timofte R. (2021). Designing a Practical Degradation Model for Deep Blind Image Super-Resolution. arXiv.

[14] Ahn N., Kang B., Sohn K.A. (2018). Fast, Accurate, and Lightweight Super-Resolution with Cascading Residual Network. arXiv.

[15] Goodfellow I.J., Pouget-Abadie J., Mirza M., Xu B., Warde-Farley D., Ozair S., Courville A., Bengio Y. (2014). Generative Adversarial Networks. arXiv.

[16] Zeng K., Zheng H., Cai C., Yang Y., Zhang K., Chen Z. (2018). Simultaneous single- and multi-contrast super-resolution for brain MRI images based on a convolutional neural network. Comput. Biol. Med..

[17] Wang L., Zhu H., He Z., Jia Y., Du J. (2022). Adjacent slices feature transformer network for single anisotropic 3D brain MRI image super-resolution. Biomed. Signal Process. Control.

[18] Park S., Gahm J.K. (2022). Super-Resolution of 3D Brain MRI With Filter Learning Using Tensor Feature Clustering. IEEE Access.

[19] Pham C.H., Tor-Díez C., Meunier H., Bednarek N., Fablet R., Passat N., Rousseau F. (2019). Multiscale brain MRI super-resolution using deep 3D convolutional networks. Comput. Med Imaging Graph..

[20] Feng C.M., Wang K., Lu S., Xu Y., Li X. (2021). Brain MRI super-resolution using coupled-projection residual network. Neurocomputing.

[21] Wu Z., Chen X., Xie S., Shen J., Zeng Y. (2023). Super-resolution of brain MRI images based on denoising diffusion probabilistic model. Biomed. Signal Process. Control.

[22] Song L., Wang Q., Liu T., Li H., Fan J., Yang J., Hu B. (2022). Deep robust residual network for super-resolution of 2D fetal brain MRI. Sci. Rep..

[23] Hongtao Z., Shinomiya Y., Yoshida S. (2020). 3D Brain MRI Reconstruction based on 2D Super-Resolution Technology. Proceedings of the 2020 IEEE International Conference on Systems, Man, and Cybernetics (SMC).

[24] Buades A., Coll B., Morel J.M. (2011). Non-Local Means Denoising. Image Process. Line.

[25] Black M., Sapiro G., Marimont D., Heeger D. (1998). Robust anisotropic diffusion. IEEE Trans. Image Process..

[26] Tomasi C., Manduchi R. Bilateral filtering for gray and color images. Proceedings of the Sixth International Conference on Computer Vision (IEEE Cat. No.98CH36271).

[27] Liu H., Yuan H., Hou J., Hamzaoui R., Gao W. (2022). PUFA-GAN: A Frequency-Aware Generative Adversarial Network for 3D Point Cloud Upsampling. IEEE Trans. Image Process..

[28] Snoek L., van der Miesen M.M., Beemsterboer T., van der Leij A., Eigenhuis A., Scholte H.S. (2021). The Amsterdam Open MRI Collection, a set of multimodal MRI datasets for individual difference analyses. Sci. Data.

[29] The Cancer Genome Atlas (TCGA) Research Network Dataset, U.S (2006). Department of Health and Human Services, National Institutes of Health, National Cancer Institute. https://portal.gdc.cancer.gov/.

[30] Liew S.L., Lo B.P., Donnelly M.R., Zavaliangos-Petropulu A., Jeong J.N., Barisano G., Hutton A., Simon J.P., Juliano J.M., Suri A. (2022). A large, curated, open-source stroke neuroimaging dataset to improve lesion segmentation algorithms. Sci. Data.

[31] Wang X., Yu K., Wu S., Gu J., Liu Y., Dong C., Qiao Y., Loy C.C. ESRGAN: Enhanced super-resolution generative adversarial networks. Proceedings of the European Conference on Computer Vision Workshops (ECCVW).

[32] Landman B.A., Huang A.J., Gifford A., Vikram D.S., Lim I.A.L., Farrell J.A., Bogovic J.A., Hua J., Chen M., Jarso S. (2011). Multi-parametric neuroimaging reproducibility: A 3-T resource study. NeuroImage.

[33] Wiki N. (2017). Downloads—NAMIC Wiki. https://www.na-mic.org/wiki/Downloads.

[34] Li W., Wang Y., Su Y., Li X., Liu A.A., Zhang Y. (2023). Multi-Scale Fine-Grained Alignments for Image and Sentence Matching. IEEE Trans. Multimed..

[35] Cong R., Sheng H., Yang D., Cui Z., Chen R. (2023). Exploiting Spatial and Angular Correlations With Deep Efficient Transformers for Light Field Image Super-Resolution. IEEE Trans. Multimed..

[36] Cheng D., Chen L., Lv C., Guo L., Kou Q. (2022). Light-Guided and Cross-Fusion U-Net for Anti-Illumination Image Super-Resolution. IEEE Trans. Circuits Syst. Video Technol..

[37] Sheng H., Wang S., Yang D., Cong R., Cui Z., Chen R. (2023). Cross-View Recurrence-based Self-Supervised Super-Resolution of Light Field. IEEE Trans. Circuits Syst. Video Technol..

[38] Essen D.C.V., Smith S.M., Barch D.M., Behrens T.E., Yacoub E., Ugurbil K. (2013). The WU-Minn Human Connectome Project: An overview. NeuroImage.

[39] Kempton M.J., Underwood T.S., Brunton S., Stylios F., Schmechtig A., Ettinger U., Smith M.S., Lovestone S., Crum W.R., Frangou S. (2011). A comprehensive testing protocol for MRI neuroanatomical segmentation techniques: Evaluation of a novel lateral ventricle segmentation method. NeuroImage.

[40] Commowick O., Istace A., Kain M., Laurent B., Leray F., Simon M., Pop S.C., Girard P., Améli R., Ferré J.C. (2018). Objective Evaluation of Multiple Sclerosis Lesion Segmentation using a Data Management and Processing Infrastructure. Sci. Rep..

[41] Cocosco C.A., Kollokian V., Kwan R.K.-S., Evans A.C. BrainWeb: Online Interface to a 3D MRI Simulated Brain Database, Neuroimage. Proceedings of the 3rd International Conference on Functional Mapping of the Human Brain.

[42] Srinivasan R. (2022). Noise: Radiology Reference Article. Radiopaedia.

[43] Smith S.M., Jenkinson M., Woolrich M.W., Beckmann C.F., Behrens T.E., Johansen-Berg H., Bannister P.R., Luca M.D., Drobnjak I., Flitney D.E. (2004). Advances in functional and structural MR image analysis and implementation as FSL. NeuroImage.

[44] (2023). FreeSurfer, An Open-Source Software Suite for Processing Human Brain MRI. https://github.com/freesurfer/freesurfer.

[45] Li H., Wang W., Wang M., Li L., Vimlund V. (2022). A review of deep learning methods for pixel-level crack detection. J. Traffic Transp. Eng..

[46] Johnson J., Alahi A., Fei-Fei L. (2016). Perceptual Losses for Real-Time Style Transfer and Super-Resolution. arXiv.

[47] Wu B., Duan H., Liu Z., Sun G. (2017). SRPGAN: Perceptual Generative Adversarial Network for Single Image Super Resolution. arXiv.

[48] Lai W.S., Huang J.B., Ahuja N., Yang M.H. Deep Laplacian Pyramid Networks for Fast and Accurate Super-Resolution. Proceedings of the 2017 IEEE Conference on Computer Vision and Pattern Recognition (CVPR).

[49] Anagun Y., Isik S., Seke E. (2019). SRLibrary: Comparing different loss functions for super-resolution over various convolutional architectures. J. Vis. Commun. Image Represent..

[50] Gatys L., Ecker A.S., Bethge M., Cortes C., Lawrence N., Lee D., Sugiyama M., Garnett R. (2015). Texture Synthesis Using Convolutional Neural Networks. Proceedings of the Advances in Neural Information Processing Systems.

[51] Gatys L.A., Ecker A.S., Bethge M. (2015). A Neural Algorithm of Artistic Style. arXiv.

[52] Simonyan K., Zisserman A. (2014). Very Deep Convolutional Networks for Large-Scale Image Recognition. arXiv.

[53] Russakovsky O., Deng J., Su H., Krause J., Satheesh S., Ma S., Huang Z., Karpathy A., Khosla A., Bernstein M. (2015). ImageNet Large Scale Visual Recognition Challenge. Int. J. Comput. Vis..

[54] Kokaram A. (2004). Practical, Unified, Motion and Missing Data Treatment in Degraded Video. J. Math. Imaging Vis..

[55] Getreuer P. (2012). Rudin-Osher-Fatemi Total Variation Denoising using Split Bregman. Image Process. Line.

[56] Donoho D.L., Johnstone I.M. (1994). Ideal spatial adaptation by wavelet shrinkage. Biometrika.

[57] Pratt W.K. (2007). Digital Image Processing: PIKS Scientific Inside.

[58] Zhang K., Li Y., Liang J., Cao J., Zhang Y., Tang H., Timofte R., Van Gool L. (2022). Practical Blind Denoising via Swin-Conv-UNet and Data Synthesis. arXiv.

[59] Zamir S.W., Arora A., Khan S., Hayat M., Khan F.S., Yang M.H. (2021). Restormer: Efficient Transformer for High-Resolution Image Restoration. arXiv.

[60] Cai Y., Hu X., Wang H., Zhang Y., Pfister H., Wei D. (2022). Learning to Generate Realistic Noisy Images via Pixel-level Noise-aware Adversarial Training. arXiv.

[61] Chen L., Chu X., Zhang X., Sun J. (2022). Simple Baselines for Image Restoration. arXiv.

[62] Bradski G. (2000). The OpenCV Library. Dr. Dobb’s J. Softw. Tools.

[63] Van der Walt S., Schönberger J.L., Nunez-Iglesias J., Boulogne F., Warner J.D., Yager N., Gouillart E., Yu T. (2014). scikit-image: Image processing in Python. PeerJ.

[64] Virtanen P., Gommers R., Oliphant T.E., Haberland M., Reddy T., Cournapeau D., Burovski E., Peterson P., Weckesser W., Bright J. (2020). SciPy 1.0: Fundamental Algorithms for Scientific Computing in Python. Nat. Methods.

[65] Hore A., Ziou D. (2010). Image Quality Metrics: PSNR vs. SSIM. Proceedings of the 2010 20th International Conference on Pattern Recognition.

[66] Kastryulin S., Zakirov J., Pezzotti N., Dylov D.V. (2022). Image Quality Assessment for Magnetic Resonance Imaging. arXiv.

[67] Zhang L., Shen Y., Li H. (2014). VSI: A Visual Saliency-Induced Index for Perceptual Image Quality Assessment. IEEE Trans. Image Process..

[68] Lin H., Hosu V., Saupe D. KADID-10k: A Large-scale Artificially Distorted IQA Database. Proceedings of the 2019 Eleventh International Conference on Quality of Multimedia Experience (QoMEX).

[69] Zhang R., Isola P., Efros A.A., Shechtman E., Wang O. (2018). The Unreasonable Effectiveness of Deep Features as a Perceptual Metric. arXiv.

[70] Lusebrink F., Mattern H., Yakupov R., Acosta-Cabronero J., Ashtarayeh M., Oeltze-Jafra S., Speck O. (2020). Comprehensive Ultrahigh Resolution Whole Brain In Vivo MRI Dataset as a Human Phantom. Sci. Data.

[71] Schreiber S., Bernal J., Arndt P., Schreiber F., Müller P., Morton L., Braun-Dullaeus R.C., Valdés-Hernández M.D.C., Duarte R., Wardlaw J.M. (2023). Brain Vascular Health in ALS Is Mediated through Motor Cortex Microvascular Integrity. Cells.

[72] Betts M.J., Perosa V., Hämmerer D., Düzel E. (2023). Healthy aging and Alzheimer’s disease. Advances in Magnetic Resonance Technology and Applications.

[73] Naji N., Wilman A. (2023). Thin slab quantitative susceptibility mapping. Magn. Reson. Med..

[74] Ladd M.E., Quick H.H., Speck O., Bock M., Doerfler A., Forsting M., Hennig J., Ittermann B., Möller H.E., Nagel A.M. (2023). Germany’s journey toward 14 Tesla human magnetic resonance. Magn. Reson. Mater. Physics Biol. Med..

[75] Mattern H., Lüsebrink F., Speck O. (2022). High-resolution structural brain imaging. Advances in Magnetic Resonance Technology and Applications.

[76] Koenig L.N., Day G.S., Salter A., Keefe S., Marple L.M., Long J., LaMontagne P., Massoumzadeh P., Snider B.J., Kanthamneni M. (2020). Select Atrophied Regions in Alzheimer disease (SARA): An improved volumetric model for identifying Alzheimer disease dementia. NeuroImage Clin..

[77] Arjovsky M., Chintala S., Bottou L. (2017). Wasserstein GAN. arXiv.

[78] Sahoo D.K., Das A., Mohanty M.N., Mishra S. (2022). Brain tumor detection using inpainting and deep ensemble model. J. Inf. Optim. Sci..

[79] Khosla M., Jamison K., Kuceyeski A., Sabuncu M.R. (2019). Ensemble learning with 3D convolutional neural networks for functional connectome-based prediction. NeuroImage.

[80] Nguyen D., Nguyen H., Ong H., Le H., Ha H., Duc N.T., Ngo H.T. (2022). Ensemble learning using traditional machine learning and deep neural network for diagnosis of Alzheimer’s disease. IBRO Neurosci. Rep..

[81] Saber M., Boulmaiz T., Guermoui M., Abdrabo K.I., Kantoush S.A., Sumi T., Boutaghane H., Hori T., Binh D.V., Nguyen B.Q. (2023). Enhancing flood risk assessment through integration of ensemble learning approaches and physical-based hydrological modeling. Geomat. Nat. Hazards Risk.

[82] Yeganeh A., Pourpanah F., Shadman A. (2021). An ANN-based ensemble model for change point estimation in control charts. Appl. Soft Comput..

